# Stronger Together: Examining the Interaction Effects of Workplace Dignity and Workplace Inclusion on Employees’ Job Performance

**DOI:** 10.3389/fpsyg.2022.891189

**Published:** 2022-05-23

**Authors:** Ammarah Ahmed, Dapeng Liang, Muhammad Adeel Anjum, Dilawar Khan Durrani

**Affiliations:** ^1^School of Management, Harbin Institute of Technology, Harbin, China; ^2^Department of Management Sciences, Balochistan University of Information Technology, Engineering and Management Sciences (BUITEMS), Quetta, Pakistan; ^3^Department of Commerce, University of Balochistan, Quetta, Pakistan

**Keywords:** workplace dignity, workplace inclusion, job performance, job demands-resources model, Pakistan

## Abstract

Despite growing interest in workplace dignity, there is a paucity of empirical research regarding whether and when it leads to higher job performance. To address these research gaps, this study examines the relationship between workplace dignity and job performance, identifying and examining the boundary condition role of workplace inclusion. Multi-source and time-lagged data were obtained from employee–supervisor dyads (*n* = 169) in non-governmental organizations in Pakistan to test the hypothesized model, employing techniques, such as confirmatory factor analysis, moderated multiple regression, post-hoc slope, and Johnson–Neyman analyses. As predicted, workplace dignity and workplace inclusion positively influenced employees’ job performance, while workplace inclusion moderated the dignity-performance relationship such that this relationship was more strongly positive when workplace inclusion was high. At the theoretical level, this study adds new insights to the job demands-resources (JD-R) model, which is used as theoretical lens in this study. Specifically, this study is the first to examine workplace dignity and its consequences from the perspective of the JD-R model, thus introducing a new theoretical perspective into the dignity literature. This study also provides useful advice for management practice, policymaking, and employees, and is germane to the United Nations’ Sustainable Development Goal 8.

## Introduction

No one can deny the importance of employee performance for organizations. It is commonly believed that organizations are only as successful as their employees and that successful employees are those who can perform their jobs well or execute assigned duties and tasks efficiently and effectively. Without employee performance, there is no unit performance, no team performance, and no organizational performance. Hence, employee performance can be considered as a fundamental building block on which organizational performance is based (Campbell and Wiernik, 2015, p. 48). Employee performance has been classified into three categories: task or in-role performance (in-role behaviors that facilitate the provision of a service or the production of a good); citizenship performance (extra-role and voluntary behaviors that help to achieve the organizational goals); and counterproductive performance (behaviors that damage the wellbeing of the organization and its members; Rotundo and Sackett, 2002, pp. 67–68). Although all facets of performance have a unique importance, this study addresses task or job performance (JP) because it is one of the most important dimensions of employee performance (Johnson, 2001; Rotundo and Sackett, 2002) that directly affects organizational performance (Almatrooshi et al., 2016). Hence, it is critical to explore factors that might contribute to employees’ JP.

Although a great deal of research has been undertaken in this regard (e.g., Carmeli and Josman, 2006; Kamdar and Van Dyne, 2007; Tsai et al., 2007; Shantz et al., 2013; Yu and Frenkel, 2013; Kim et al., 2015; Du et al., 2016; Bodla and Ningyu, 2017; Yang and Wei, 2017; Chen and Tang, 2018; Khoreva and Wechtler, 2018; Khalid, 2020; Lin et al., 2020; Kim and Kim, 2021; Tran et al., 2021), the JP literature has some notable gaps. For instance, research has yet to look into the links between bright-side aspects of organizational life, for example, workplace dignity (WD), and employees’ JP (Chen and Tang, 2018). The first objective of this study is to address this gap in the literature. Specifically, this study investigates the effects of WD [“the value or worth that individuals acquire from work” (Lucas, 2017, p. 2549)] on employees’ JP. Further, factors that might accentuate the performance effects of WD remain unknown. To address this gap, the current study examines the boundary condition role of workplace inclusion (WI), defined as “the extent to which employee feel that they belong to and are socially included in the workplace” (Pearce and Randel, 2004, p. 84) in the link between WD and JP. In examining these perspectives, this study adds the following insights to the literature.

First, while most research on WD is qualitative (e.g., Hodson and Roscigno, 2004; Sayer, 2007; Yalden and McCormack, 2010; Lucas, 2011, 2015; Crowley, 2013, 2014; Lucas et al., 2013; Baker and Lucas, 2017; Noronha et al., 2020; King et al., 2021; Tiwari et al., 2021), this study is a pioneering empirical work explaining and examining the link between WD and JP, thus expanding the scant body of empirical research investigating WD’s relationship with employee behaviors (Thomas and Lucas, 2019; Ahmed et al., 2021; Wang et al., 2021). Second, this study furthers our understanding of the circumstances/conditions in which WD is more likely to enhance employees’ JP. It is worth mentioning the lack of research identifying and examining the factors that might interact with WD and accentuate its effects. Hence, the importance of this study cannot be ignored. Third, there has been equivocal evidence regarding the relationship between WI and JP; for example, while Cho and Mor Barak (2008) and Pearce and Randel (2004) observed a statistically significant relationship between these constructs, Chen and Tang (2018) found no association between them (*r* = 0.12, *p* > 0.05). The present study aims to reduce this ambiguity by examining the WI-JP relationship. Finally, at the theoretical level, this study seeks to add new insights to the job demands-resources (JD-R) model (Bakker and Demerouti, 2007), which is used as theoretical lens in this study. This study is a maiden attempt to ascertain the relationship dynamics between WD, WI, and JP from the perspective of the JD-R model, thus introducing a new theoretical perspective into the WD literature and extending the applicability of the JD-R model. This study contends that WD and WI are job resources that positively influence JP and that the interaction of job resources (WD and WI) leads to positive outcomes. To test these assumptions, not only does this study introduce new job resources, it also provides valuable insights regarding the interaction between job resources, which is rare in the literature. In addition to informing the literature, this study is important for management practice in that it can help HR managers to understand the extent to which WD and WI are beneficial for organizations, and to develop interventions *via* which dignity and inclusion can be bolstered. Organizational policymakers/decision-makers can also utilize this study’s findings to craft/modify organizational policies.

The remainder of this article is structured as follows. Following this introduction, the theoretical foundations and hypotheses are presented, followed by the methodology. The results are then presented and subsequently discussed. Following sections on implications (for policy, management practice, and employees) and limitations and directions for future research, the final conclusions are presented.

## Theoretical Foundations and Hypotheses

This study draws on the JD-R model (Bakker and Demerouti, 2007) and relevant empirical works to explicate the WD–JP relationship and the boundary condition role of WI. The JD-R model states that job resources (e.g., factors, such as autonomy and support) have motivational potential and can facilitate the attainment of positive organizational and work-related outcomes (e.g., excellent JP and higher work engagement; Bakker and Demerouti, 2007). Since WD has a motivational potential and it can lead to several positive outcomes, such as higher levels of work effort and work engagement, as well as increased propensity to display citizenship behaviors (Thomas and Lucas, 2019; Ahmed et al., 2021; Wang et al., 2021), we contend that it is a job resource that can positively influence employees’ JP. Similarly, based on the findings that WI is functional in attaining several desirable outcomes (e.g., organizational commitment, team member role performance, innovator role, and JP; Pearce and Randel, 2004; Chen and Tang, 2018), we submit that WI is also a job resource that can enhance employees’ JP. Further, using insights from relevant empirical works showing that job resources can interact to predict positive outcomes (Ahmed et al., 2020), we postulate that WI might enhance the performance effects of WD, that is, the WD-JP relationship will be stronger positive when WI is high. The theoretical reasoning for these assumptions is explicated in the following subsections.

### Workplace Dignity and Job Performance

Dignity is a phenomenon that has been described and conceptualized in multiple ways (Mitchell, 2017; Teixeira et al., 2021). In some cases, it has been viewed as a psychological outcome that can be felt, realized, and pursued (expressed by using metaphors, such as a “sense of dignity”), while in other instances, it has been referred to as the quality of human interactions, which can be maintained, improved, or even harmed. Regardless of how it is perceived and described, dignity remains salient to human beings in all walks of life (Lucas, 2015). Although dignity has been an important part of the scholarly discourse throughout history (for a review and discussion, see Bal, 2017; Lucas, 2017; Thomas and Lucas, 2019), it has emerged as a theoretically distinct construct in the work domain based largely on the seminal work of Randy Hodson, which not only introduced a general definition of dignity but also highlighted conditions/factors that may threaten it. According to Hodson (2001, p. 3), dignity is an individual’s ability to establish her/his self-worth, and to appreciate and recognize the respect of others. Bal (2017, p. 73) presented a different perspective, considering WD as “the intrinsic and unalienable worth of everything (e.g., human and non-human elements) in the workplace.” He argued that dignity is not limited only to people; the workplace and its non-human elements also have their own dignity. Therefore, dignity in the workplace and its elements should be upheld (Bal, 2017). However, since Bal’s (2017) conceptualization of WD entails elements that are not the focus of this study (non-human elements in the workplace), we use Kristen Lucas’s more relevant definition of WD, that is, “the self and others’ acknowledged worth acquired from engaging in work activity” (Lucas, 2017, p. 2549). As a construct, WD is composed of multiple dimensions (e.g., respectful interactions, equality, and inherent value). Four main principles are important to understand it: (i) WD is communicatively bound and depends upon individuals’ self-evaluation of their own worth and how others acknowledge this worth; (ii) the nature of WD is self-construed, that is, individuals are the ultimate arbiters of their experiences of dignity in the workplace; (iii) WD entails both unconditional and conditional sources of worth; and (iv) WD has a bivalent nature, that is, one cannot understand and experience dignity without attending to its absence (Thomas and Lucas, 2019).

As a construct, WD is related to, but distinct from, the constructs that entail elements of self-worth and/or self-esteem [e.g., organization-based self-esteem (OBSE) and organizational respect]. WD differs from OBSE in that it also embodies other-recognized worth and does not function along a single continuum (i.e., low and high; Thomas and Lucas, 2019, p. 102). Empirical research has shown that WD and OBSE are two different constructs (Ahmed et al., 2021). Similarly, WD differs from organizational respect in terms of its scope, that is, while organizational respect emphasizes only on the giving of respect within an organization (Ramarajan et al., 2008), WD entails several other factors, such as expressions of inherent value and equality, as well as the recognition of others’ competence and contributions (Thomas and Lucas, 2019). This incremental validity of WD has been established by Thomas and Lucas (2019), who found that WD explained considerable variance in several variables above and beyond the predictive effects of organizational respect. Similarly, WD differs from decent work (Duffy et al., 2017) in that it is an outcome of the latter (Scott-Campbell and Williams, 2020). In simple words, decent work serves as a means by which WD is upheld or violated. Although a comprehensive review of how WD differs from other related constructs in management is beyond this study’s scope, it is noteworthy that WD is theoretically different from constructs, such as integrity, fairness, organizational justice, value, and equality (for a discussion, see Bal, 2017; King et al., 2021). Having explained WD and relevant theoretical foundations in detail, let us now turn the discussion to how this might affect employees’ JP (the central theme of this research). Before proceeding to this discussion, a brief delineation of the eminent conceptualizations of this construct is required. According to Campbell et al. (1990), performance consists of those observable job-related behaviors that are pertinent to the organizational objectives and goals. Motowidlo et al. (1997), meanwhile, held that performance, rather than solely being a behavioral manifestation, also has an evaluative aspect, that is, the overall value that employees’ behaviors might carry. Consistent with these conceptualizations, the current study defines employees’ JP in terms of their in-role contributions (Williams and Anderson, 1991).

In line with the premise that job resources have motivational potential and that job resources can lead to positive outcomes (e.g., higher levels of JP; Bakker and Demerouti, 2007, p. 313), this study predicts that, as a job resource, WD will positively influence employees’ JP. Although no empirical evidence is available regarding the WD-JP relationship, research has demonstrated that WD can foster employees’ wellbeing and lead to certain desirable outcomes, such as increased work engagement, high OBSE, more discretionary work effort, and increased propensity to display citizenship behaviors (Lucas et al., 2017; Thomas and Lucas, 2019; Ahmed et al., 2021; Teixeira et al., 2021; Wang et al., 2021). Using insights from these empirical works, we contend that WD may positively influence employees’ JP. Research has also highlighted that employees’ sense of ownership in work, as well as their inclination to perform work, might increase if they are treated in a dignified manner in the workplace (Hodson, 2001). Hence, we propose that:

*Hypothesis 1*: Employees’ perceptions of WD will be positively associated with their JP.

### The Moderating Role of Workplace Inclusion

Similar to WD, WI has been defined in multiple ways (Chen and Tang, 2018; Shore et al., 2018). For instance, while Mor Barak and Cherin (1998) defined WI in terms of employees’ perceptions regarding the extent to which they are a part of important organizational processes (e.g., decision-making) and have access to valuable resources, Pelled et al. (1999) referred it to as the degree to which employees are accepted and treated as insiders in the workplace. Some scholars have even defined WI from the perspective of leadership, workgroups, work environment, and organizational practices (for a review, see Shore et al., 2018). Consistent with Chen and Tang (2018), this study defines WI in terms of employees’ perceptions regarding the extent to which they belong to, and are socially included in, the workplace (Pearce and Randel, 2004, p. 84). WI is often confused with workplace diversity. However, these are two different constructs. While diversity is concerned mainly with bringing people of different color, race, religion, nationality, ethnicity, social and sexual identity, and gender into the workplace, inclusion seeks to create opportunities through which such individuals can take part, influence important organizational processes, and utilize organizational resources. Scholars have argued that, while diversity is easier to achieve (e.g., diversity can be achieved through legislation or mandatory practices), a great deal of effort and voluntarism is required to establish and/or enhance WI, that is, ensuring real prospects of equal access to valued opportunities for everyone in the workplace is not easy and requires inputs from all organizational members. Further, it is worth noting that diversity does not always lead to beneficial outcomes; for example, the differences among people may increase interpersonal conflicts and adversely affect group cohesion. However, such consequences are less likely when the workplace is inclusive (Shore et al., 2018, pp. 177–178). In fact, inclusion is the means through which both the problems and benefits associated with diversity can be managed (Shore et al., 2011; Nishii, 2013).

Let us now explicate the relationship between WI and JP. We argue that WI can motivate employees to perform well. The main reason why WI seems likely to positively influence employees’ JP is that when employees recognize themselves in the organizational mainstream or feel included, they tend to work harder and perform well (Pearce and Randel, 2004). This is mainly because WI facilitates the exchange of information and allows employees to develop specialized skills (e.g., networking and work flexibly) and knowledge that can improve their efficiency and performance (e.g., the information regarding work practices and procedures may enable employees to be quicker and more effective in completing their tasks). Further, employees working in inclusive workplaces are more likely to subordinate their personal objectives and goals to the needs of the organization and work devotedly, thus performing better (Pearce and Randel, 2004). Further, experiencing inclusion can induce several positive feelings in employees (e.g., feeling valued and supported; Chen and Tang, 2018), which may bolster their performance. Hence, it can be expected that higher levels of WI can lead to greater JP. This postulation receives direct support from past empirical works indicating a positive association between employees’ perceptions of WI and their JP (e.g., Pearce and Randel, 2004; Cho and Mor Barak, 2008). Hence, we propose that:

*Hypothesis 2*: Employees’ perceptions of WI will be positively associated with their JP.

In addition to bolstering employees’ JP, WI may also increase the positive effects of WD on JP. We postulate this based on past empirical works suggesting that the interaction between job resources can lead to positive outcomes. Specifically, Ahmed et al. (2020) found that a strong service climate strengthens the positive effects of developmental HR practices (e.g., training and development and career development opportunities) on employees’ work engagement. These authors suggested that the advantages of developmental HR practices can be brought out more when the service climate is stronger. In a similar vein, we contend that the performance advantage of WD is amplified when WI is high. Therefore, we propose that:

*Hypothesis 3*: WI will moderate the positive relationship between WD and JP such that this relationship will be stronger when WI is high than when it is low.

**Figure 1:**
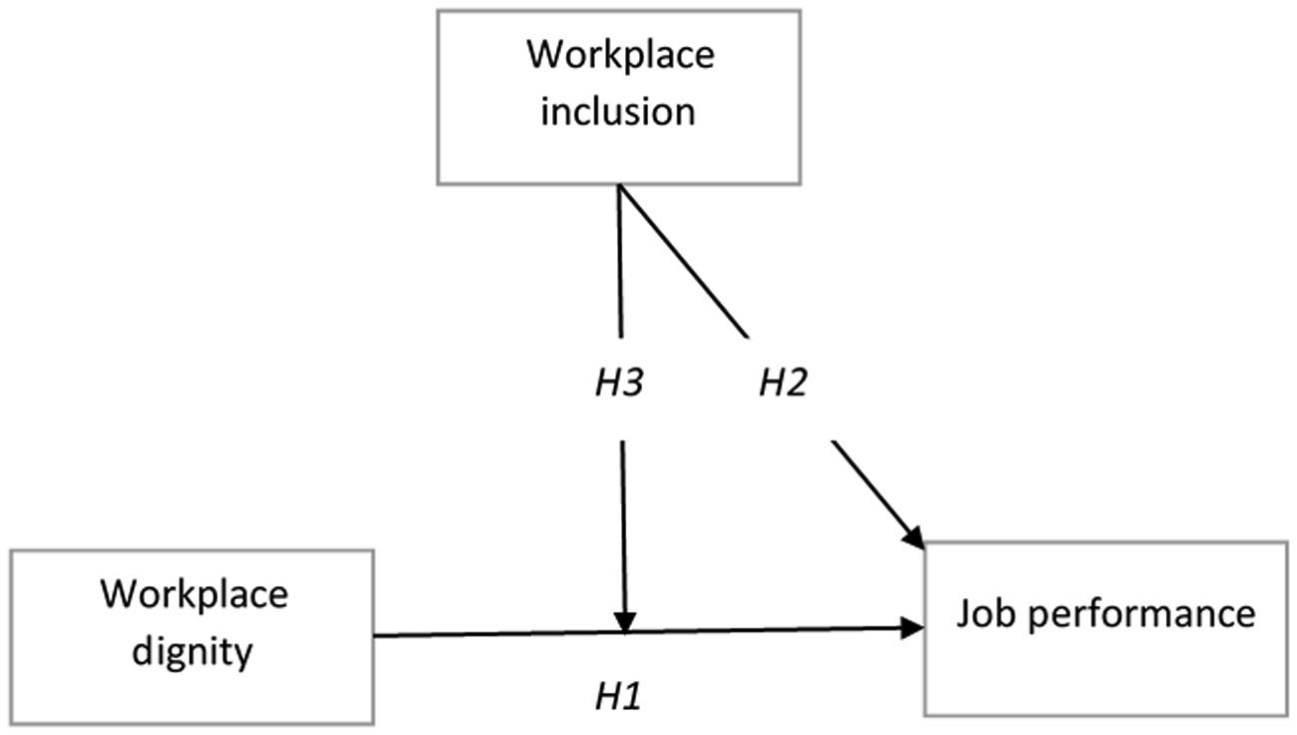
Hypothesized research model.

The framework for this study is displayed in Figure 1.

## Materials and Methods

### Context, Design, and Sample

Context can play an important role in understanding WD and its consequences (Lee, 2008; Tiwari and Sharma, 2019), that is, WD may be perceived and experienced differently in Western (individualistic) and non-Western (collectivistic) cultures. In Western cultures, for example, people are inherently entitled to dignity in the workplace; therefore, they may not value it consciously until it is abused or harmed. However, dignity may be perceived much more consciously in non-Western cultures because it is usually not an inherent privilege of employment in such cultures. Research conducted in non-Western cultures (e.g., China and Pakistan) has also indicated that WD can have profound effects on employees’ work attitudes and behaviors (Ahmed et al., 2021; Wang et al., 2021). Hence, the venue for this study (i.e., Pakistan) is suitable for examining the effects of WD.

Data for this study were obtained from the employee-supervisor dyads in non-governmental organizations (NGOs) based in the capital city of the Balochistan province in Pakistan because these organizations have been argued to be suitable for examining the consequences of WD (for discussion, see Ahmed et al., 2021). To avoid problems associated with a cross-sectional research design and single-source data [e.g., the inability to make causal inferences, and common method bias (CMB) or common method variance; Podsakoff et al., 2003], a time-lagged research design and a multi-source data collection approach were adopted. Specifically, data were gathered in two phases, with a time lag/interval of three weeks, to minimize the likelihood that performance-affecting events might occur in between the assessment of employees’ perceptions of WD and WI and their JP (De Clercq et al., 2019) using a structured paper-based survey that comprised two parts: an employee survey that contained an informed consent form, questions on demographic variables, and battery of questions on WD and WI; and a supervisor survey of employees’ JP. A cover letter with all necessary details (e.g., the introduction to this research and instructions on how to respond) accompanied both the surveys.

Leveraging personal and professional contacts, existing employees of the NGOs (*N* = 226) were approached at time 1 (T1) and requested to participate in part one of this study’s surveys. Following Ahmed et al. (2021) and Anjum et al. (2021a), two attention check questions [“Please encircle yes to indicate that you are paying attention” (Scott-Campbell and Williams, 2020) and “Please encircle agree for this question” (Kung et al., 2018)] were included in the employees’ survey to ensure obtaining quality data. Further, respondents were requested to provide information regarding their employee code, job title, and department in the demographic profile section of the survey. This information was included in the introductory part of the JP survey to facilitate employees’ identification and obtain their performance ratings from the supervisors. To ensure confidentiality and minimize the likelihood of social desirability bias, surveys were distributed and collected by the researchers in blank/unmarked envelops.

Phase one of the data collection generated an initial sample of 182 surveys, of which 13 were discarded for incomplete information (no employee codes or incorrect answers to the attention check questions). In phase two, supervisory ratings for 169 employees (those who provided the requisite information and passed the attention check) were obtained. Hence, the final sample size (*n*) for this study was 169 (final response rate = 74.77%), surpassing our *a-priori* estimation of 138 participants to detect medium effect size [power_(1–*β*)_ = 0.95; error probability = 0.05; predictors = 05 (Faul et al., 2009)]. The descriptive analysis (Table 1) indicated that the average age of participants was 32.350 years (*SD* = 5.826), and the majority were male (*n* = 108). The education-wise classification of participants was as follows: undergraduate = 10 (5.9%); graduate = 79 (46.7%); master’s = 64 (37.9%); and others = 16 (9.5%).

**Table 1:** Descriptive statistics.

Variables	Mean	SD	*n*	Percentage
Gender				
Male	**–**	**–**	108	63.9
Female	**–**	**–**	61	36.1
Age	32.350	5.826	**–**	**–**
Education				
-Graduation	**–**	**–**	79	46.7
-Masters	**–**	**–**	64	37.9
-Other	**–**	**–**	16	09.5
WD	3.769	0.787	**–**	**–**
WI	3.942	0.741	**–**	**–**
JP	4.061	0.745	**–**	**–**

*SD = standard deviation; n = frequency*.

### Measures

Because English is the official language of almost all organizations in Pakistan (De Clercq et al., 2019; Jahanzeb et al., 2020; Anjum et al., 2021b), the English versions of the following measures, with five response options (1 = “strongly disagree”; 5 = “strongly agree”), were used:

*Workplace dignity:* Consistent with indigenous research (Ahmed et al., 2021), participants’ perceptions of WD were measured using 14 items (e.g., “My workplace is a source of dignity for me”; Thomas and Lucas, 2019). Similar to the reliability coefficient of α = 0.96 obtained by Thomas and Lucas (2019), the reliability coefficient for this scale was found to be 0.937.

*Workplace inclusion:* Following Chen and Tang (2018), participants’ perceptions of WI were assessed using a three-item scale (e.g., “I feel included in most activities at work”; Pearce and Randel, 2004). The scale had high internal consistency/reliability (*α* = 0.813).

*Job performance:* Similar to De Clercq et al. (2019), supervisory ratings of employees’ JP were obtained using a seven-item in-role behaviors scale (e.g., “This employee adequately completes assigned duties”; Williams and Anderson, 1991). The reliability coefficient for this scale was 0.899, which is similar to that of Jahanzeb et al. (2020; *α* = 0.87).

### Control Variables

Consistent with previous empirical works on JP (Wheeler et al., 2012; Chen and Tang, 2018; De Clercq et al., 2019; Jahanzeb et al., 2020), the effects of respondents’ gender (male = 1, female = 2), education (undergraduate = 1, graduate = 2, master’s = 3, and others = 4), and age were controlled because these variables might affect JP. For instance, there is a possibility that the knowledge gains associated with higher levels of education might enhance employees’ capabilities in meeting performance standards, or women might perform their jobs more diligently than men (De Clercq et al., 2019, p. 191).

## Analaysis and Results

Before proceeding to hypotheses testing, a series of confirmatory factor analysis (CFA) were run to check whether the data fitted the hypothesized measurement model and to compute the parameters of construct validity (discriminant/divergent and convergent validity). For this, Analysis of Moment Structures (AMOS) software (version 23) was used. As Table 2 demonstrates, the three-factor model (WD, WI, and JP) had superior model fitting (χ^2^/df = 1.124, *p* > 0.05; GFI = 0.882; IFI = 0.986; TLI = 0.984; CFI = 0.985; RMSEA = 0.027; PClose>0.05) than the alternate models with different collapsing combinations of variables. Despite the fact that our data were multi-source and time-lagged, two additional CFA models (a CFA model in which all survey items were forced to represent a single factor, and a CFA model in which all survey items, in addition to their respective latent factor, were allowed to load on an unmeasured latent factor) were also run to determine the extent of CMB in the data. The single-factor model had a poor fit with the data. Similarly, the common latent factor (CLF) model fitted less well with the data as compared with the hypothesized measurement model, suggesting that the data for this study have no serious CMB problems (Bagozzi and Yi, 1990; Anjum et al., 2021b).

**Table 2:** Goodness of fit analysis.

Model	χ^2^/df (*value of p*)	GFI	IFI	TLI	CFI	RMSEA	PClose
Three-factor model	1.124(0.087)	0.882	0.986	0.984	0.985	0.027	0.995
Three-factor CLF model	1.150(0.061)	0.890	0.984	0.980	0.984	0.030	0.987
Two-factor model^a^	1.632(0.000)	0.831	0.926	0.918	0.925	0.061	0.045
Two-factor model^b^	1.823(0.000)	0.810	0.904	0.893	0.902	0.070	0.001
Two-factor model^c^	3.009(0.000)	0.614	0.765	0.738	0.762	0.109	0.000
Single-factor model	3.494(0.000)	0.591	0.706	0.675	0.703	0.122	0.000

*χ^2^ = chi-square; df = degrees of freedom; GFI = goodness of fit index; TLI = Tucker–Lewis index; CFI = comparative fit index; RMSEA = root mean square approximation of error; CLF = common latent factor.*

a Two-factor model = WD + WI, JP.

b Two-factor model=WD, WI + JP.

c Two-factor model=WD + JP, WI.

The statistics produced from testing the three-factor model (standardized item loadings and factor correlations) were further utilized to compute the parameters of construct validity [e.g., average shared variance (ASV) and average variance extracted (AVE)] using the following formulas: ASV = ∑*r^2^/n*; and AVE = ∑*λ^2^/n* (Hair et al., 2010). As Table 3 indicates, the AVE scores for all constructs (WD = 0.518, WI = 0.598, JP = 0.565) did not fall below the suggested value of 0.50, and were greater than the corresponding ASV scores, indicating adequate convergent and discriminant validity, respectively (Hair et al., 2010). For rigor, the square root values of the AVE scores (see bold values in parenthesis on the diagonal in Table 3) were also computed and compared with the correlations among variables. These values did not exceed the inter-construct correlations, providing additional support for discriminant validity (Fornell and Larcker, 1981). The composite reliability (CR) scores for all scales were also found to be greater than the suggested value of 0.70 (Hair et al., 2010), further supporting the internal consistency of the scales used. As expected, respondents’ WD and WI perceptions were positively related to supervisor-rated JP (WD and JP: *r* = 0.397, *p* < 0.01; WI and JP: *r* = 0.271, *p* < 0.01). A moderate positive association was also observed between WD and WI (WD and JP: *r* = 0.451, *p* < 0.01). Of the control variables, only gender had a statistically significant relationship with JP (*r* = 0.203, *p* < 0.01).

**Table 3:** Inter-construct correlations, reliability, and validity analyses.

Constructs	Gender	Age	Education	WD_T1_	WI_T1_	JP_T2_	AVE	ASV	CR
Gender	**–**						**–**	**–**	**–**
Age	0.013	**–**					**–**	**–**	**–**
Education	−0.116	0.358^**^	**–**				**–**	**–**	**–**
WD_T1_	0.076	−0.050	0.070	**(0.720)**			0.518	0.180	0.937
WI_T1_	−0.047	0.012	0.038	0.451^**^	**(0.773)**		0.598	0.138	0.816
JP_T2_	0.203^**^	−0.077	0.032	0.397^**^	0.271^**^	**(0.753)**	0.565	0.115	0.901

*M = mean; bold values in diagonal are square root of AVE; CR = Composite reliability.*

** *p < 0.01*.

Using the Statistical Package for Social Sciences (SPSS, version 21) and the PROCESS macro for SPSS (Hayes, 2018), moderated multiple regression and slope analyses were performed to test the hypotheses. Following the guidelines by Aiken and West (1991), the predictor (WD) and moderator (WI) variables were mean-centered and an interaction term (WD × WI) was created to perform moderation analysis (see Table 4). Similar to De Clercq et al. (2019), only gender (Male = 1; Female = 2) had a statistically significant relationship with JP (Model 1: *B* = 0.334, *p* < 0.05; Model 2: *B* = 0.300, *p* < 0.05; Model 3: *B* = 0.298, *p* < 0.05), while education (Model 1: *B* = 0.098, *p* > 0.05; Model 2: *B* = 0.058, *p* > 0.05; Model 3: *B* = 0.058, *p* > 0.05) and age (Model 1: *B* = -0.015, *p* > 0.05; Model 2: *B* = -0.011, *p* > 0.05; Model 3: *B* = -0.011, *p* > 0.05) had non-significant relationships with JP. As the results indicate, WD and WI had significant positive relationships with JP (WD➔JP: *B* = 0.353, *p* < 0.05; WI➔JP: *B* = 0.196, *p* < 0.05), supporting Hypothesis 1 and Hypothesis 2. The interaction term (Model 3) was also found to be statistically significant, thus Hypothesis 3 was supported. To clarify the interaction effects, a slope analysis (Table 5) was conducted (Aiken and West, 1991), which indicated that the WD-JP relationship was more strongly positive when WI was high (+1SD: *B* = 0.469, *p* < 0.05) than when WI was low (-1SD: *B* = 0.237, *p* < 0.05). Since the WD-JP relationship was significant both at ±1SD of WI, Johnson–Neyman analysis was run to further probe the interaction effects (Hayes, 2018). Results of this additional analysis (Table 6) highlighted that the WD-JP relationship ceased to be significant at low values of the moderator (e.g., -1.2761) and was more strongly positive at high values of the moderator, supporting our stance that the performance effects of WD can be brought out more when WI is high than when it is low.

**Table 4:** Moderation analysis.

Predictors and model statistics	Outcome variable: JP		
	Model 1	Model 2	Model 3
	*B*	*B*	*B*
*Control variables* Gender Education Age *Predictor variables* WD WI WD × WI	0.334^**^ 0.098 −0.015	0.300^**^ 0.058 −0.011 0.294^**^ 0.140^**^	0.289^**^ 0.058 −0.011 0.353^**^ 0.196^**^ 0.156^**^
*Model statistics* *F* *R^2^* Adjusted *R^2^* *R^2^* change	3.249^**^ 0.056 0.039 **–**	8.600^**^ 0.209 0.184 0.153	8.130^**^ 0.231 0.203 0.023

*JP = job performance; B = unstandardized coefficient; WD×WI = interaction term*.

** *p < 0.05*.

**Table 5:** Slope analysis.

Level of moderator	*B*	*t*	95% CI	
			LB	UB
Low (−1 *SD*)	0.237	3.023^**^	0.082	0.392
High (+1 *SD*)	0.469	4.301^**^	0.254	0.684

*CI = confidence interval; LB = lower bound of 95% CI; UB = upper bound of 95% CI; SD = standard deviation*.

** *p < 0.05*.

**Table 6:** The Johnson–Neyman analysis of interaction effects.

WI	*B*	*se*	*t*	*p*	LB	UB
−2.109	0.023	0.144	0.164	0.869	−0.260	0.308
−1.942	0.049	0.134	0.371	0.710	−0.214	0.314
−1.776	0.075	0.124	0.609	0.542	−0.169	0.321
−1.609	0.101	0.114	0.885	0.377	−0.125	0.328
−1.442	0.127	0.106	1.204	0.230	−0.081	0.337
−1.276	0.153	0.097	1.570	0.118	−0.039	0.347
−1.113	0.179	0.090	1.974	0.050	0.000	0.358
−1.109	0.179	0.090	1.984	0.048	0.000	0.358
−0.942	0.205	0.084	2.441	0.015	0.039	0.372
−0.776	0.231	0.079	2.922	0.004	0.075	0.388
−0.609	0.257	0.075	3.398	0.000	0.108	0.407
−0.442	0.283	0.074	3.825	0.000	0.137	0.430
−0.276	0.309	0.074	4.163	0.000	0.162	0.456
−0.109	0.335	0.076	4.389	0.000	0.184	0.486
0.057	0.361	0.080	4.505	0.000	0.203	0.520
0.223	0.387	0.085	4.529	0.000	0.218	0.557
0.390	0.413	0.092	4.490	0.000	0.231	0.595
0.557	0.439	0.099	4.411	0.000	0.243	0.636
0.723	0.465	0.108	4.312	0.000	0.252	0.679
0.890	0.491	0.117	4.205	0.000	0.260	0.722
1.057	0.517	0.126	4.097	0.000	0.268	0.767

**Figure 2:**
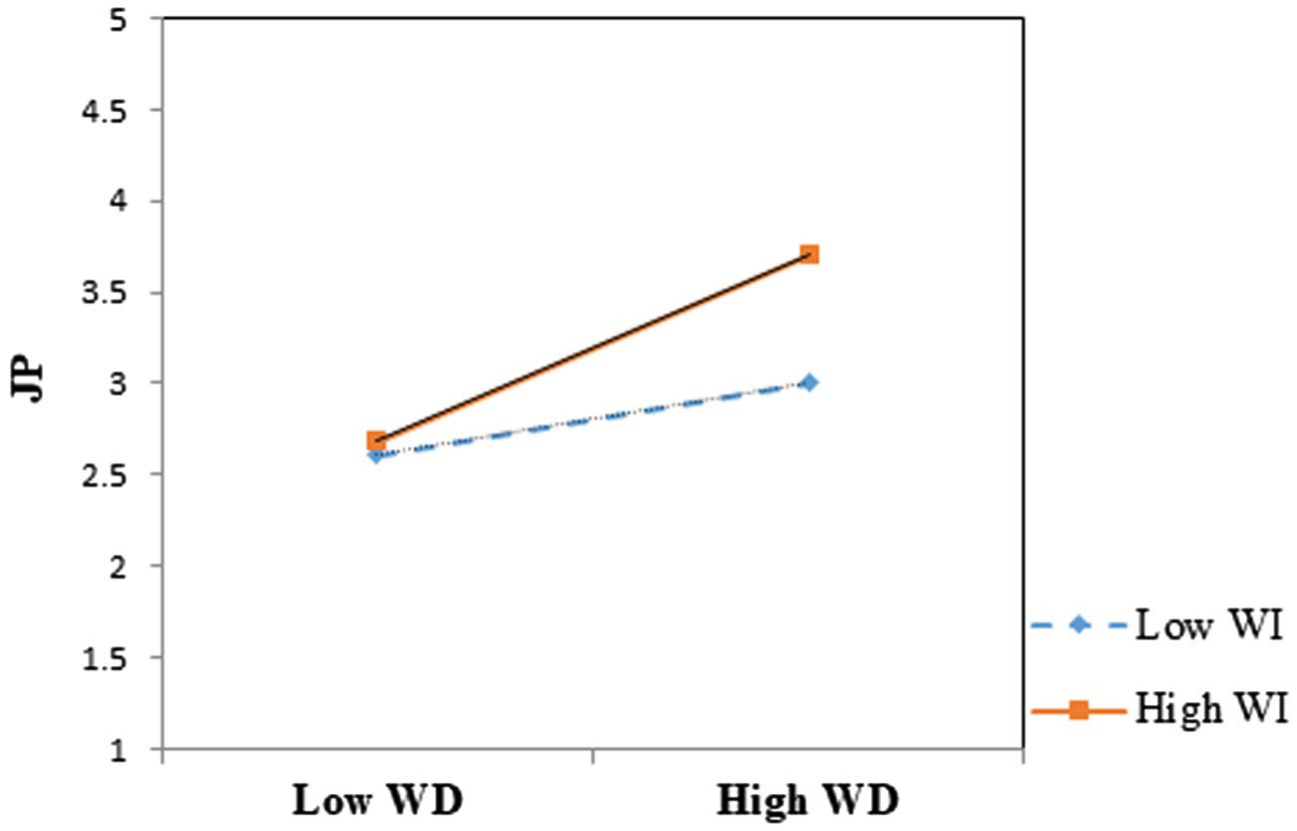
The moderation effects of workplace inclusion.

The interaction effects are further explicated in Figure 2, which confirms that WI strengthened the positive relationship between WD and JP. Following Becker (2005) and De Clercq et al. (2019), the analysis was rerun without certain control variables that had a non-significant effect on JP and consistent results were obtained.

## Discussion

This study’s aim was to answer the following broad questions: “Does WD affect employees’ JP?” and “When is WD more likely to affect employees’ JP?” Specifically, the WD-JP relationship, taking into account the moderating role of WI, was examined in this study. Using insights from relevant empirical works and the JD-R model, this study postulated that WD would positively affect employees’ JP (Hypothesis 1). The data supported this hypothesis, and a statistically significant positive relationship was found between WD and JP. This finding is consistent with both the argument that WD is an important cause of employee behaviors (Lucas et al., 2017) and empirical research showing positive associations between WD and employee behaviors (e.g., work engagement, work effort, and organizational citizenship behaviors; Lucas et al., 2017; Thomas and Lucas, 2019; Ahmed et al., 2021; Wang et al., 2021). The positive relationship between WD and JP can be attributed to the key features/attributes of the former; for example, recognition of one’s competence and contribution, respectful interactions, and expressions of worth and equality in the workplace may motivate employees to perform well. In other words, the positive feelings and/or attitudes evoked by WD can boost employees’ performance. Although there has been no study on the direct relationship between WD and JP, the strength of these constructs’ relationship (*r* = 0.397; *B* = 0.353) is similar to that found by Ahmed et al. (2021), indicating that WD is a powerful organizational phenomenon that plays a pivotal role in explaining employee behaviors (Lucas et al., 2017; Thomas and Lucas, 2019). The finding also supports our postulation that WD is an important job resource that can translate to bottom-line organizational objectives (e.g., increased performance). Hence, it could be asserted that experiencing WD is not only good for employees, but is also an important condition for organizations in that it fosters employee behaviors that positively affect organizational performance and effectiveness (Lucas et al., 2017).

Consistent with Cho and Mor Barak (2008) and Pearce and Randel (2004), a positive relationship was observed between WI and JP. Using insights from the JD-R model (Bakker and Demerouti, 2007) and past empirical research (Pearce and Randel, 2004; Cho and Mor Barak, 2008; Chen and Tang, 2018), we theorized that WI is a job resource that positively influences employees’ JP. WI’s statistically significant positive association with JP provided empirical support to this notion. Interestingly, the strength of WI-JP relationship (*r* = 0.271) is similar to that found by Pearce and Randel (2004; *r* = 0.26) and Cho and Mor Barak (2008; *r* = 0.244), supporting the argument that higher levels of WI can foster performance at the individual level (Pearce and Randel, 2004; Shore et al., 2011). This finding also attests to our postulation that WI is functional in attaining work-related outcomes. Past research has also shown employees’ perceptions of inclusion to be positively linked to important outcomes, such as commitment to the organization, satisfaction with job, and task performance (Cho and Mor Barak, 2008; Nishii, 2013; Brimhall et al., 2017; Chen and Tang, 2018). Hence, WI can be considered a job resource. The positive association between WI and JP signifies that working in an inclusive workplace is a psychologically positive experience that encourages employees to perform better in given roles. In other words, employees’ feelings that they are recognized, valued, and socially integrated in the organization can increase their performance (Cho and Mor Barak, 2008; Chen and Tang, 2018). In summary, our findings reflect that job resources have an intrinsic motivational potential (Xanthopoulou et al., 2009). For example, WD’s and WI’s positive associations with JP demonstrate that employees who receive dignified treatment in the workplace and feel included in the corporate mainstream are intrinsically motivated and therefore perform better. Hence, we conclude that when job resources are available for employees, they demonstrate greater performance. As shown in Table 2, a positive association was also observed between WD and WI, reinforcing the findings that job resources tend to correlate with each other (Xanthopoulou et al., 2007, 2009).

Finally, as predicted in Hypothesis 3, WI moderated the WD-JP relationship such that this relationship was more strongly positive when WI was high than when it was low, suggesting that WI is an important organizational factor that bolsters the performance outcomes of WD. Although the moderation effect of WI on the WD-JP relationship has not been examined previously, the finding attests to the notions that inclusion plays a crucial role in organizational life (Mor Barak et al., 2001; Mor Barak and Levin, 2002; Nishii, 2013; Shore et al., 2018), and that the interaction between job resources leads to positive outcomes (Ahmed et al., 2020). Based on our finding regarding Hypothesis 3, it may be concluded that WI is a favorable organizational condition under which the performance benefits of job resources are strengthened and enhanced.

## Implications for Policymaking, Management Practice, and Employees

Highlighting the importance of WI and WD in the workplace, this study offers several insights for management practice and policymaking. As the results indicate, WI and WD contribute to employees’ performance; therefore, policymakers and HR managers should devise policies and implement efforts that promote dignity and inclusion in the workplace. Similar to leading companies globally (e.g., Ernst and Young and BASF), organizational policymakers in Pakistan should endeavor to establish an inclusive organizational culture, in which every organizational member, irrespective of his/her gender, color, race, religion, or rank in the organizational hierarchy, is valued, fairly treated, and included in critical/core organizational processes (Chen and Tang, 2018). Policymakers are also suggested to declare dignity as a core value of organizational culture (Ahmed et al., 2021). Further, the business-level strategies and HR policies should be formulated/revised in such a way that they do not compromise employees’ inclusion and dignity. For instance, decisions to relocate, deploy, and develop employees should be taken after a proper consultation with, and input from, all stake stakeholders, especially those for whom such decisions are being taken. This will make everyone feel that they are included in the workplace. Similarly, employees’ dignity should not be sacrificed while taking critical organizational decisions (e.g., redundancies; Bal and de Jong, 2017; Ahmed et al., 2021). In summary, inclusion and dignity should be the key focus of all organizational policies (Shore et al., 2011; Bal and de Jong, 2017).

Apart from the policy recommendations noted above, this study offers useful advice for HR managers/practitioners regarding how employees’ dignity can be protected and WI can be ensured. First of all, HR managers must address factors that could make employees perceive that they are socially excluded in the workplace and that their dignity is at risk in the organization (e.g., weak or fluctuating organizational culture, low task interdependence, disrespectful communication, a high-stress work environment, abusive supervision, reification, incivility, and bullying; Robinson et al., 2013; Lucas et al., 2017). Having addressed such factors, HR managers/practitioners should make conscious efforts to bolster dignity and facilitate inclusion through various organizational practices and processes. Leadership is one such factor/process that can play a significant role in this regard; for example, leaders’ behaviors, such as showing respect for employees, acknowledging their competence and contribution, and involving them in (and seeking input/suggestions on) important matters, can foster inclusion and dignity in the workplace. Research has also highlighted that leadership can play a key role in increasing inclusion and dignity. For instance, Brimhall et al. (2017) found that the quality of a leader’s relationship with employees can increase inclusiveness in an organization. Similarly, Omari (2010) argued that organizational leadership can ameliorate dignity issues. Therefore, due attention should be paid to developing effective leadership skills (Brooks and Chapman, 2018) for those individuals in supervisory and/or leadership roles. Similarly, organizational practices that satisfy employees’ need for belongingness, uniqueness, and self-esteem should be implemented to foster inclusion (Shore et al., 2011) and dignity. In summary, a strategic and integrated approach should be followed to ensure inclusion and dignity in the workplace (Omari, 2010; Bal and de Jong, 2017; Ahmed et al., 2021).

Finally, employees, who are the ultimate arbiters of inclusion and dignity, should play their part to enhance inclusion and dignity in the workplace. Some small steps, such as protecting the honor and dignity of one’s colleagues, valuing their opinions and preferences, acknowledging mutual differences, giving others a compassionate ear during difficult times, and including everyone in professional camaraderie can have a significant effect.

## Limitations and Directions for Future Research

Despite its contributions, this study has some limitations, which should be noted. First, data for this study were gathered from NGOs, which have a unique culture and workplace dynamic. Therefore, the findings obtained may not be directly applicable to other sectors and/or industries. Hence, this study should be replicated in other sectors and industries. Second, despite the fact that the research model developed and tested herein is novel and theoretically sound, it is narrow in scope, that is, certain mechanisms that might underlie and/or affect the WD–JP relationship were not measured because the main goal of this study was to ascertain only whether WD influences employees’ in-role behaviors. Therefore, future researchers should identify and examine the mediating mechanisms of the WD-JP relationship. One possible causal mechanism that might link dignity with performance is motivation [a psychological force that gives behaviors a direction and purpose (Kreitner, 1995)]. The mediating role of motivation is consistent with the motivation process of the JD-R model, which pinpoints that job resources lead to desirable outcomes through motivation (Bakker and Demerouti, 2007). Hence, this perspective could be considered in future studies. Similarly, certain organizational factors that might enhance the effects of WD on JP; for example, perceived organizational support (Rhoades and Eisenberger, 2002) and civility climate (Walsh et al., 2012) could also be examined.

Further, WD’s effects on outcomes, such as employees’ workability (McGonagle et al., 2015), creativity (Amabile and Pratt, 2016), citizenship behaviors (Podsakoff et al., 2000), and job embeddedness (Mitchell et al., 2001), could also be examined as empirical research examining the consequences of experiencing WD is particularly scant (Lucas et al., 2017; Thomas and Lucas, 2019). Third, since WD may not be perceived particularly consciously in Western cultures, it can be speculated that the results (e.g., the strength of the WD-JP relationship) might differ if this study were replicated/conducted in a Western context. Since research examining variability in how employees from different cultures perceive and react to WD does not exist, cross-cultural studies are strongly recommended. Fourth, despite receiving empirical support, this study’s notion that WD and WI are job resources requires further research. Fifth, although multi-source and time-lagged data were collected, the causality of the tested relationships remains limited. Moreover, supervisory ratings of performance, as obtained in this study, might also contain bias (Werner, 1994). These issues may be addressed in continued research by following robust experimental research designs (lab or field experiments with treatment and control groups). Finally, besides quantitative studies, more in-depth research approaches (e.g., mixed-methods studies) may be adopted to study the phenomenon of WD and its consequences.

## Conclusion

By corroborating the relationship dynamics among WD, WI, and JP, this study highlights the importance of dignity and inclusion in workplaces. Results from this study indicate that WD is an important and impactful organizational phenomenon that can positively predict employees’ in-role behaviors. Therefore, due efforts should be made to ensure and bolster dignity in the workplace. Results also highlight that WI is an important organizational factor in that it fosters employee performance and enhances the performance effects of WD; hence, conscious efforts should be made to promote inclusion in organizations.

## Data Availability Statement

The raw data supporting the conclusions of this article will be made available by the authors, without undue reservation.

## Ethics Statement

Ethical review and approval was not required for the study on human participants in accordance with the local legislation and institutional requirements. The patients/participants provided their written informed consent to participate in this study.

## Author Contributions

All authors listed have made a substantial, direct, and intellectual contribution to the work, and approved it for publication.

## Conflict of Interest

The authors declare that the research was conducted in the absence of any commercial or financial relationships that could be construed as a potential conflict of interest.

## Publisher’s Note

All claims expressed in this article are solely those of the authors and do not necessarily represent those of their affiliated organizations, or those of the publisher, the editors and the reviewers. Any product that may be evaluated in this article, or claim that may be made by its manufacturer, is not guaranteed or endorsed by the publisher.

## Appendix

### Further Reading

Bolton, S. C. (2007). “Dignity in and at work: why it matters,” in *Dimensions of Dignity at Work*. ed. S. C. Bolton (Oxford, UK: Butterworth-Heinemann), 3–16. doi: 10.1016/B978-0-7506-8333-3.50006-8

Hodson, R. (1996). Dignity in the workplace under participative management: alienation and freedom revisited. *Am. Sociol. Rev.* 61, 719–738. doi: 10.2307/2096450

Hodson, R. (2007). “The consequences of management competence for working with dignity: A cross-methods validation,” in *Dimensions of Dignity at Work*. ed. S. C. Bolton (Oxford, UK: Butterworth-Heinemann), 109–133. doi: 10.1016/B978-0-7506-8333-3.50012-3

Islam, G. (2013). Recognizing employees: reification, dignity and promoting care in management. *Cross Cultural Management: An International Journal* 20, 235–250. doi: 10.1108/13527601311313490

Lamont, M. (2000). *The Dignity of Working Men*, Cambridge, MA: Harvard University Press, doi: 10.4159/9780674039889

Pirson, M. (2017). *Humanistic Management: Protecting Dignity and Promoting Wellbeing*. Cambridge, UK: Cambridge University Press, doi: 10.1017/9781316675946

Zawadzki, M. (2018). Dignity in the workplace: the perspective of humanistic management. *J. Manag. Business Admin. Central Europe* 26, 171–188. doi: 10.7206/jmba.ce.2450-7814.224

## References

[ref1] Ahmed U. Kura K. M. Umrani W. A. Pahi M. H. (2020). Modelling the link between developmental human resource practices and work engagement: the moderation role of service climate. Glob. Bus. Rev. 21, 31–53. doi: 10.1177/0972150919837813

[ref2] Ahmed A. Liang D. Anjum M. A. Durrani D. K. (2021). Does dignity matter? The effects of workplace dignity on organization-based self-esteem and discretionary work effort. Current Psychol. 1–12. doi: 10.1007/s12144-021-01821-5 [Epub ahead of print]

[ref3] Aiken L. S. West S. G. (1991). Multiple Regression: Testing and Interpreting Interactions. Newbury Park, CA: Sage Publications, Inc.

[ref4] Almatrooshi B. Singh S. K. Farouk S. (2016). Determinants of organizational performance: a proposed framework. Int. J. Product. Perform. Manag. 65, 844–859. doi: 10.1108/IJPPM-02-2016-0038

[ref5] Amabile T. M. Pratt M. G. (2016). The dynamic componential model of creativity and innovation in organizations: making progress, making meaning. Res. Organ. Behav. 36, 157–183. doi: 10.1016/j.riob.2016.10.001

[ref6] Anjum M. A. Ahmed A. Zhang L. Durrani D. K. (2021a). How rude! Linking supervisor incivility to subordinates’ discretionary work effort. Int. J. Confl. Manag 32, 867–885. doi: 10.1108/IJCMA-04-2021-0054

[ref7] Anjum M. A. Liang D. Ahmed A. Parvez A. (2021b). Understanding how and when workplace ostracism jeopardizes work effort. Manag. Decis. doi: 10.1108/MD-02-20210195 [Epub ahead of print]

[ref8] Bagozzi R. P. Yi Y. (1990). Assessing method variance in multitrait-multimethod matrices: the case of self-reported affect and perceptions at work. J. Appl. Psychol. 75, 547–560. doi: 10.1037/0021-9010.75.5.547

[ref9] Baker S. J. Lucas K. (2017). Is it safe to bring myself to work? Understanding LGBTQ experiences of workplace dignity. Canadian J. Adm. Sci 34, 133–148. doi: 10.1002/cjas.1439

[ref10] Bakker A. B. Demerouti E. (2007). The job demands-resources model: state of the art. J. Manag. Psychol. 22, 309–328. doi: 10.1108/02683940710733115

[ref11] Bal M. (2017). Dignity in the Workplace: New Theoretical Perspectives, Springer: Berlin.

[ref12] Bal P. M. de Jong S. B. (2017). “From human resource management to human dignity development: a dignity perspective on HRM and the role of workplace democracy” in Dignity and the Organization. eds. Kostera M. Pirson M. (London: Palgrave Macmillan), 173–195.

[ref13] Becker T. E. (2005). Potential problems in the statistical control of variables in organizational research: a qualitative analysis with recommendations. Organ. Res. Methods 8, 274–289. doi: 10.1177/1094428105278021

[ref14] Bodla A. A. Ningyu T. (2017). Transformative HR practices and employee task performance in high-tech firms: The role of employee adaptivity. J. Organ. Chang. Manag. 30, 710–724. doi: 10.1108/JOCM-02-2016-0030

[ref15] Brimhall K. C. Mor Barak M. E. Hurlburt M. McArdle J. J. Palinkas L. Henwood B. (2017). Increasing workplace inclusion: The promise of leader-member exchange. Human Service Organ. Manag. Leadership Governance 41, 222–239. doi: 10.1080/23303131.2016.1251522

[ref16] Brooks B. Chapman N. H. (2018). Leadership is learned. J. Leadersh. Stud. 12, 72–74. doi: 10.1002/jls.21582 29864235

[ref17] Campbell J. P. McHenry J. J. Wise L. L. (1990). Modeling job performance in a population of jobs. Pers. Psychol. 43, 313–575. doi: 10.1111/j.1744-6570.1990.tb01561.x

[ref18] Campbell J. P. Wiernik B. M. (2015). The modeling and assessment of work performance. Annu. Rev. Organ. Psych. Organ. Behav. 2, 47–74. doi: 10.1146/annurev-orgpsych-032414-111427

[ref19] Carmeli A. Josman Z. E. (2006). The relationship among emotional intelligence, task performance, and organizational citizenship behaviors. Hum. Perform. 19, 403–419. doi: 10.1207/s15327043hup1904_5

[ref20] Chen C. Tang N. (2018). Does perceived inclusion matter in the workplace? J. Manag. Psychol. 33, 43–57. doi: 10.1108/JMP-02-2017-0078

[ref21] Cho S. Mor Barak M. E. (2008). Understanding of diversity and inclusion in a perceived homogeneous culture: a study of organizational commitment and job performance among Korean employees. Adm. Soc. Work. 32, 100–126. doi: 10.1080/03643100802293865

[ref22] Crowley M. (2013). Gender, the labor process and dignity at work. Soc. Forces 91, 1209–1238. doi: 10.1093/sf/sot042

[ref23] Crowley M. (2014). Class, control, and relational indignity: labor process foundations for workplace humiliation, conflict, and shame. Am. Behav. Sci. 58, 416–434. doi: 10.1177/0002764213503335

[ref24] De Clercq D. Haq I. U. Azeem M. U. (2019). Workplace ostracism and job performance: roles of self-efficacy and job level. Pers. Rev. 48, 184–203. doi: 10.1108/PR-02-2017-0039

[ref25] Du Y. Zhang L. Chen Y. (2016). From creative process engagement to performance: bidirectional support. Leadership Organ. Develop. J 37, 966–982. doi: 10.1108/LODJ-03-2015-0046

[ref26] Duffy R. D. Allan B. A. England J. W. Blustein D. L. Autin K. L. Douglass R. P.. (2017). The development and initial validation of the decent work scale. J. Couns. Psychol. 64, 206–221. doi: 10.1037/cou0000191, PMID: 28165257

[ref27] Faul F. Erdfelder E. Buchner A. Lang A.-G. (2009). Statistical power analyses using G*power 3.1: tests for correlation and regression analyses. Behav. Res. Methods 41, 1149–1160. doi: 10.3758/BRM.41.4.1149, PMID: 19897823

[ref28] Fornell C. Larcker D. F. (1981). Evaluating structural equation models with unobservable variables and measurement error. J. Mark. Res. 18, 39–50. doi: 10.1177/002224378101800104

[ref29] Hair J. F. Black W. C. Babin B. J. Anderson R. E. (2010). Multivariate Data Analysis: A Global Perspective, Upper Saddle River, NJ Prentice Hall: Pearson.

[ref30] Hayes A. F. (2018). Introduction to Mediation, Moderation, and Conditional Process Analysis: A Regression-Based Approach, 2nd Edn., Guilford Publications, New York, NY.

[ref31] Hodson R. (2001). Dignity at Work, Cambridge: Cambridge University Press, doi: 10.1017/CBO9780511499333

[ref32] Hodson R. Roscigno V. J. (2004). Organizational success and worker dignity: complementary or contradictory? Am. J. Sociol. 110, 672–708. doi: 10.1086/422626

[ref33] Jahanzeb S. Fatima T. Javed B. Giles J. P. (2020). Can mindfulness overcome the effects of workplace ostracism on job performance? J. Soc. Psychol. 160, 589–602. doi: 10.1080/00224545.2019.1707465, PMID: 31870244

[ref34] Johnson J. W. (2001). The relative importance of task and contextual performance dimensions to supervisor judgments of overall performance. J. Appl. Psychol. 86, 984–996. doi: 10.1037/0021-9010.86.5.984, PMID: 11596814

[ref35] Kamdar D. Van Dyne L. (2007). The joint effects of personality and workplace social exchange relationships in predicting task performance and citizenship performance. J. Appl. Psychol. 92, 1286–1298. doi: 10.1037/0021-9010.92.5.1286, PMID: 17845086

[ref36] Khalid K. (2020). The impact of managerial support on the association between pay satisfaction, continuance and affective commitment, and employee task performance. SAGE Open 1–13. doi: 10.1177/2158244020914591

[ref37] Khoreva V. Wechtler H. (2018). HR practices and employee performance: the mediating role of well-being. Empl. Relat. 40, 227–243. doi: 10.1108/ER-08-2017-0191

[ref38] Kim M. Kim J. (2021). Corporate social responsibility, employee engagement, well-being and the task performance of frontline employees. Manag. Decis. 59, 2040–2056. doi: 10.1108/MD-03-2020-0268

[ref39] Kim T. Y. Liu Z. Diefendorff J. M. (2015). Leader–member exchange and job performance: the effects of taking charge and organizational tenure. J. Organ. Behav. 36, 216–231. doi: 10.1002/job.1971

[ref40] King O. Davis C. Clemans A. Coles J. Crampton P. Jacobs N.. (2021). Dignity during work-integrated learning: what does it mean for supervisors and students? Stud. High. Educ. 46, 721–736. doi: 10.1080/03075079.2019.1650736

[ref41] Kreitner R. (1995). Management, 6th Edn., Boston: Houghton Mifflin Company.

[ref42] Kung F. Y. Kwok N. Brown D. J. (2018). Are attention check questions a threat to scale validity? Appl. Psychol. 67, 264–283. doi: 10.1111/apps.12108

[ref43] Lee M. Y. K. (2008). Universal human dignity: Some reflections in the Asian context. Asian J. Comparative Law 3, 283–313. doi: 10.2202/1932-0205.1076

[ref44] Lin C. P. Huang H. T. Huang T. Y. (2020). The effects of responsible leadership and knowledge sharing on job performance among knowledge workers. Pers. Rev. 49, 1879–1896. doi: 10.1108/PR-12-2018-0527

[ref45] Lucas K. (2011). Blue-collar discourses of workplace dignity: using outgroup comparisons to construct positive identities. Manag. Commun. Q. 25, 353–374. doi: 10.1177/0893318910386445

[ref46] Lucas K. (2015). Workplace dignity: communicating inherent, earned, and remediated dignity. J. Manag. Stud. 52, 621–646. doi: 10.1111/joms.12133

[ref47] Lucas K. (2017). “Workplace dignity,” in The International Encyclopedia of Organizational Communication. eds. Scott C. R. Lewis L. (Wiley Blackwell: Chichester, UK), 2549–2562. doi: 10.1002/9781118955567.wbieoc222

[ref48] Lucas K. Kang D. Li Z. (2013). Workplace dignity in a total institution: examining the experiences of Foxconn’s migrant workforce. J. Bus. Ethics 114, 91–106. doi: 10.1007/s10551-012-1328-0

[ref49] Lucas K. Manikas A. S. Mattingly E. S. Crider C. J. (2017). Engaging and misbehaving: how dignity affects employee work behaviors. Organ. Stud. 38, 1505–1527. doi: 10.1177/0170840616677634

[ref50] McGonagle A. K. Fisher G. G. Barnes-Farrell J. L. Grosch J. W. (2015). Individual and work factors related to perceived work ability and labor force outcomes. J. Appl. Psychol. 100, 376–398. doi: 10.1037/a0037974, PMID: 25314364 PMC4879965

[ref51] Mitchell L. (2017). “Dignity and membership: a route to the heart of how dignity is done in everyday interaction,” in Dignity and the Organization. eds. Kostera M. Pirson M. (London, England: Palgrave Macmillan).

[ref52] Mitchell T. R. Holtom B. C. Lee T. W. Sablynski C. J. Erez M. (2001). Why people stay: using job embeddedness to predict voluntary turnover. Acad. Manag. J. 44, 1102–1121. doi: 10.5465/3069391

[ref53] Mor Barak M. E. Cherin D. A. (1998). A tool to expand organizational understanding of workforce diversity: exploring a measure of inclusion-exclusion. Adm. Soc. Work. 22, 47–64. doi: 10.1300/J147v22n01_04

[ref54] Mor Barak M. E. Findler L. Wind L. H. (2001). Diversity, inclusion, and commitment to organizations: international empirical explorations. J. Behav. Appl. Manag. 2, 70–91.

[ref55] Mor Barak M. E. Levin A. (2002). Outside of the corporate mainstream and excluded from the work community: a study of diversity, job satisfaction and well-being. Community Work Fam. 5, 133–157. doi: 10.1080/13668800220146346

[ref56] Motowidlo S. J. Borman W. C. Schmit M. J. (1997). A theory of individual differences in task and contextual performance. Hum. Perform. 10, 71–83. doi: 10.1207/s15327043hup1002_1

[ref57] Nishii L. H. (2013). The benefits of climate for inclusion for gender-diverse groups. Acad. Manag. J. 56, 1754–1774. doi: 10.5465/amj.2009.0823

[ref58] Noronha E. Chakraborty S. D’Cruz P. (2020). Doing dignity work: Indian security guards’ interface with precariousness. J. Bus. Ethics 162, 553–575. doi: 10.1007/s10551-018-3996-x

[ref59] Omari M. (2010). Towards dignity and respect at work: an exploration of work behaviours in a professional environment, Available at http://www.aphref.aph.gov.au/house/committee/ee/bullying/subs/sub130_a.pdf

[ref60] Pearce J. L. Randel A. E. (2004). Expectations of organizational mobility, workplace social inclusion, and employee job performance. J. Organ. Behav. 25, 81–98. doi: 10.1002/job.232

[ref61] Pelled L. H. Ledford G. E. Jr. Mohrman S. A. (1999). Demographic dissimilarity and workplace inclusion. J. Manag. Stud. 36, 1013–1031. doi: 10.1111/1467-6486.00168

[ref62] Podsakoff P. M. MacKenzie S. B. Lee J. Y. Podsakoff N. P. (2003). Common method biases in behavioral research: a critical review of the literature and recommended remedies. J. Appl. Psychol. 88, 879–903. doi: 10.1037/0021-9010.88.5.879, PMID: 14516251

[ref63] Podsakoff P. M. MacKenzie S. B. Paine J. B. Bachrach D. G. (2000). Organizational citizenship behaviors: a critical review of the theoretical and empirical literature and suggestions for future research. J. Manag. 26, 513–563. doi: 10.1177/014920630002600307

[ref64] Ramarajan L. Barsade S. G. Burack O. R. (2008). The influence of organizational respect on emotional exhaustion in the human services. J. Posit. Psychol. 3, 4–18. doi: 10.1080/17439760701750980

[ref65] Rhoades L. Eisenberger R. (2002). Perceived organizational support: a review of the literature. J. Appl. Psychol. 87, 698–714. doi: 10.1037/0021-9010.87.4.698 12184574

[ref66] Robinson S. L. O’Reilly J. Wang W. (2013). Invisible at work: an integrated model of workplace ostracism. J. Manag. 39, 203–231. doi: 10.1177/0149206312466141

[ref67] Rotundo M. Sackett P. R. (2002). The relative importance of task, citizenship, and counterproductive performance to global ratings of job performance: a policy-capturing approach. J. Appl. Psychol. 87, 66–80. doi: 10.1037/0021-9010.87.1.66, PMID: 11916217

[ref68] Sayer A. (2007). Dignity at work: broadening the agenda. Organization 14, 565–581. doi: 10.1177/1350508407078053

[ref69] Scott-Campbell C. Williams M. (2020). Validating the workplace dignity scale, Collabra. Psychology 6, 1–15. doi: 10.1525/collabra.337

[ref70] Shantz A. Alfes K. Truss C. Soane E. (2013). The role of employee engagement in the relationship between job design and task performance, citizenship and deviant behaviours. Int. J. Hum. Resour. Manag. 24, 2608–2627. doi: 10.1080/09585192.2012.744334

[ref71] Shore L. M. Cleveland J. N. Sanchez D. (2018). Inclusive workplaces: a review and model. Hum. Resour. Manag. Rev. 28, 176–189. doi: 10.1016/j.hrmr.2017.07.003

[ref72] Shore L. M. Randel A. E. Chung B. G. Dean M. A. Holcombe Ehrhart K. Singh G. (2011). Inclusion and diversity in work groups: a review and model for future research. J. Manag. 37, 1262–1289. doi: 10.1177/0149206310385943

[ref73] Teixeira M. L. M. das Graças Torres Paz M. Alves S. S. (2021). “Dignity and well-being at work,” in Organizational Dignity and Evidence-Based Management. eds. Teixeira M. L. M. Oliveira L. M. B. (Springer, Cham), 271–281.

[ref74] Thomas B. Lucas K. (2019). Development and validation of the workplace dignity scale. Group Org. Manag. 44, 72–111. doi: 10.1177/1059601118807784

[ref75] Tiwari A. Sharma R. R. (2019). Dignity at the workplace: evolution of the construct and scale development. Front. Psychol. 10:2581. doi: 10.3389/fpsyg.2019.02581, PMID: 31849740 PMC6895210

[ref76] Tiwari A. Sharma T. Sharma R. R. (2021). Exploring workplace dignity from managerial lens. Manag. Res. Rev. 45, 545–562. doi: 10.1108/MRR-08-2020-0544

[ref77] Tran L. T. T. Hien H. T. V. Baker J. (2021). When supportive workplaces positively help work performance. Balt. J. Manag. 16, 208–227. doi: 10.1108/BJM-06-2020-0220

[ref78] Tsai W.-C. Chen C.-C. Liu H.-L. (2007). Test of a model linking employee positive moods and task performance. J. Appl. Psychol. 92, 1570–1583. doi: 10.1037/0021-9010.92.6.1570 18020797

[ref79] Walsh B. M. Magley V. J. Reeves D. W. Davies-Schrils K. A. Marmet M. D. Gallus J. A. (2012). Assessing workgroup norms for civility: the development of the civility norms questionnaire-brief. J. Bus. Psychol. 27, 407–420. doi: 10.1007/s10869-011-9251-4

[ref80] Wang D. Baker M. A. Kim Y. S. Ma E. (2021). From angels to demons: uncovering the relationships between tipping, social dignity, OCB and incivility intentions. Int. J. Hosp. Manag. 98:103043. doi: 10.1016/j.ijhm.2021.103043

[ref81] Werner J. M. (1994). Dimensions that make a difference: examining the impact of in-role and extra-role behaviors on supervisory ratings. J. Appl. Psychol. 79, 98–107. doi: 10.1037/0021-9010.79.1.98

[ref82] Wheeler A. R. Harris K. J. Sablynski C. J. (2012). How do employees invest abundant resources? The mediating role of work effort in the job-embeddedness/job-performance relationship. J. Appl. Soc. Psychol. 42, E244–E266. doi: 10.1111/j.1559-1816.2012.01023.x

[ref83] Williams L. J. Anderson S. E. (1991). Job satisfaction and organizational commitment as predictors of organizational citizenship and in-role behavior. J. Manag. 17, 601–617. doi: 10.1177/014920639101700305

[ref84] Xanthopoulou D. Bakker A. B. Demerouti E. Schaufeli W. B. (2007). The role of personal resources in the job demands-resources model. Int. J. Stress. Manag. 14, 121–141. doi: 10.1037/1072-5245.14.2.121

[ref85] Xanthopoulou D. Bakker A. B. Demerouti E. Schaufeli W. B. (2009). Reciprocal relationships between job resources, personal resources, and work engagement. J. Vocat. Behav. 74, 235–244. doi: 10.1016/j.jvb.2008.11.003

[ref86] Yalden B. J. McCormack B. (2010). Constructions of dignity: a pre-requisite for flourishing in the workplace? Int. J. Older People Nursing 5, 137–147. doi: 10.1111/j.1748-3743.2010.00218.x, PMID: 20925715

[ref87] Yang Q. Wei H. (2017). Ethical leadership and employee task performance: examining moderated mediation process. Manag. Decis. 55, 1506–1520. doi: 10.1108/MD-09-2016-0627

[ref88] Yu C. Frenkel S. J. (2013). Explaining task performance and creativity from perceived organizational support theory: which mechanisms are more important?, J. Organ. Behav., 34 Placeholder Text, 1165–1181. doi: 10.1002/job.1844

